# Restoration of Altered MicroRNA Expression in the Ischemic Heart with Resveratrol

**DOI:** 10.1371/journal.pone.0015705

**Published:** 2010-12-23

**Authors:** Partha Mukhopadhyay, Subhendu Mukherjee, Kaimul Ahsan, Angshuman Bagchi, Pal Pacher, Dipak K. Das

**Affiliations:** 1 Laboratory of Physiologic Studies, National Institute on Alcohol Abuse and Alcoholism, National Institute of Health, Bethesda, Maryland, United States of America; 2 Buck Institute for Age Research, Novato, California, United States of America; 3 Cardiovascular Research Center, University of Connecticut School of Medicine, Farmington, Connecticut, United States of America; Brigham and Women's Hospital, United States of America

## Abstract

**Background:**

Resveratrol, a constituent of red wine, is important for cardioprotection. MicroRNAs are known regulators for genes involved in resveratrol-mediated cardiac remodeling and the regulatory pathway involving microRNA has not been studied so far.

**Methods:**

We explored the cardioprotection by resveratrol in ischemia/reperfusion model of rat and determined cardiac functions. miRNA profile was determined from isolated RNA using quantitative Real-time PCR based array. Systemic analyses of miRNA array and theirs targets were determined using a number of computational approaches.

**Results:**

Cardioprotection by resveratrol and its derivative in ischemia/reperfusion [I/R] rat model was examined with miRNA expression profile. Unique expression pattern were found for each sample, particularly with resveratrol [pure compound] and longevinex [commercial resveratrol formulation] pretreated hearts. Longevinex and resveratrol pretreatment modulates the expression pattern of miRNAs close to the control level based on PCA analyses. Differential expression was observed in over 25 miRNAs, some of them, such as miR-21 were previously implicated in cardiac remodeling. The target genes for the differentially expressed miRNA include genes of various molecular function such as metal ion binding, sodium-potassium ion, transcription factors, which may play key role in reducing I/R injury.

**Conclusion:**

Rats pretreated with resveratrol for 3 weeks leads to significant cardioprotection against ischemia/reperfusion injury. A unique signature of miRNA profile is observed in control heart pretreated with resveratrol or longevinex. We have determined specific group of miRNA in heart that have altered during IR injuries. Most of those altered microRNA expressions modulated close to their basal level in resveratrol or longevinex treated I/R mice.

## Introduction

Resveratrol is a naturally occurring phytoalexin belonging to stilbene family of compounds and its IUPAC (International Union of Pure and Applied Chemistry) nomenclature is 3,4′,5-trihydroxystilbene. The most commonly found trans isomer of resveratrol possesses greater biological activity due to the presence of the 4′-hydroxystyryl group. Although resveratrol was first found as an anti-proliferative agent for cancer [1], overwhelming epidemiological and experimental evidence documents resveratrol as a cardioprotective agent, which supports the concept of red wine for maintaining a healthy heart [2]. It is increasingly clear that resveratrol is beneficial against diverse cardiac diseases including ischemic heart disease, hypertrophy, heart failure, atherosclerosis, hypertension, diabetes and obesity [3], [4]. In addition to being cardioprotective, resveratrol has been found to be beneficial against a variety of degenerative diseases including lung disease, alzheimer disease, hepatotoxicity, radiation damage, herpes virus [4], [5]. Resveratrol also have protective role in endothelial cells by modulating mitochondrial oxidative stress [6].

Initially, resveratrol was believed to function as an antioxidant. However resveratrol is a poor antioxidant *in vitro, but in vivo* resveratrol functions as a potent antioxidant presumably through its ability to up-regulate nitric oxide [7]. The most prominent mechanism of action is probably through its ability to perform intracellular signaling and altering gene expression. Resveratrol can alter a variety of genes thereby changing the “death signal” into a “survival signal” [8]. The most prominent mechanism appears its ability to induce several longevity genes including *Sirt1*, *Sirt3*, *Sirt4*, *FoxO1*, *Foxo3a* and *PBEF* [4]. Thus resveratrol prevents aging-related decline in cardiovascular function including cholesterol level and inflammatory response without affecting actual survival or life span of mice [4].

Since the cardioprotective abilities of resveratrol are intimately linked to the regulation of genes, it appears to be important to understand how resveratrol-mediated cardiac gene expression is controlled at the level of transcriptional regulation where transcription factors are associated with their regulatory DNA elements. The discovery of miRNAs has enhanced our understanding of gene expression at the post-transcriptional level. MicroRNAs are endogeneous small RNAs (less than 25 nt) that play key regulatory roles targeting mRNA for specific cleavage, mRNA deadenylation and decay, translational repression or activation [9], [10]. It has been shown that a number of miRNAs are expressed in tissue specific manner and contribute in various biological process like differentiation, immune modulation [11], fibrosis [12], [13] as well as in diseases like cancer [14], [15], neuro-degeneration [16] etc. Recently, miRNA has been implicated various cardiovascular development, complications and disease [17], [18], [19], [20], [21]. In response to physiological stimuli or pathological condition, such as hypertension, ischemic myocardial injury, the myocardium reacts with rapid alteration in the gene expression profile. This adaptation usually results in cardiac remodeling, which is characterized by severe structural changes of myocardial tissue, modification of the extracellular matrix, and reshaping of left ventricle geometry and performance. However, this process is only an initial “adaptive” response, which leads to development of long term damage and cardiac dysfunction. Recently, several reports have revealed important roles of miRNAs in cardiac hypertrophic growth and heart failure. We here describe for the first time miRNA expression profiling of ischemic rat hearts in the context of pre-treatments with pure resveratrol and a commercially available resveratrol formulation, longevinex, using quantitative real-time PCR based array. Furthermore, we have searched for the functional target of the key miRNA using computational analyses.

## Results and Discussion

### Resveratrol and longevinex improve cardiac function and reduce myocardial infarct size and cardiomyocyte apoptosis in the IR rat heart

**Figure 1:**
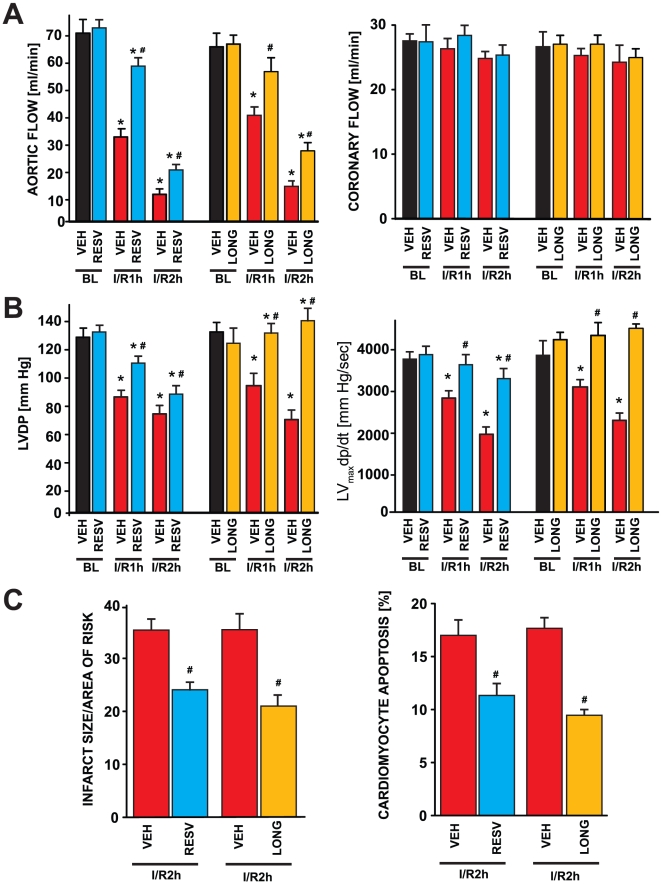
Effects of resveratrol and longevinex on blood flow, LVDP, dp/dt_max_, infarct size and apoptosis. (A) Coronary flow, aortic flow and LVDP were estimated at baseline and at the indicated times of reperfusion. Infarct size and apoptosis were measured at the end of two hours of reperfusion. Results are expressed as Means±SEM of six animals per group. *p<0.05 vs. Vehicle (VEH). # p,0.05 vs corresponding I/R. BL: Baseline; I/R1h: Ischemia for 30 min and 1 h reperfusion; I/R2h: Ischemia for 30 min and 2 h reperfusion; RESV: Resveratrol; LONG: Longevinex.

In accordance with previous studies [22], both resveratrol and longevinex improved cardiac output function including aortic flow, coronary flow, left ventricular developed pressure (LVDP) and its first derivative LV_max_dp/dt in a 2 hour of reperfusion period (Fig. 1). These compounds also lowered the infarct size and death due to cardiomyocyte apoptosis, as expected. A significant number of studies exist in the literature demonstrating cardioprotective role of resveratrol. Recent studies also showed that commercially available resveratrol formulation longevinex was equally cardioprotective. We compared the effects of resveratrol with longevinex, because recent studies determined longevinex to be equally cardioprotective without exhibiting hormetic action of resveratrol [23]. The cardioprotective effects of resveratrol and longevinex were consistent with the previously published reports [23].

### Global miRNA expression profiling in ischemia-reperfused rat heart

**Figure 2:**
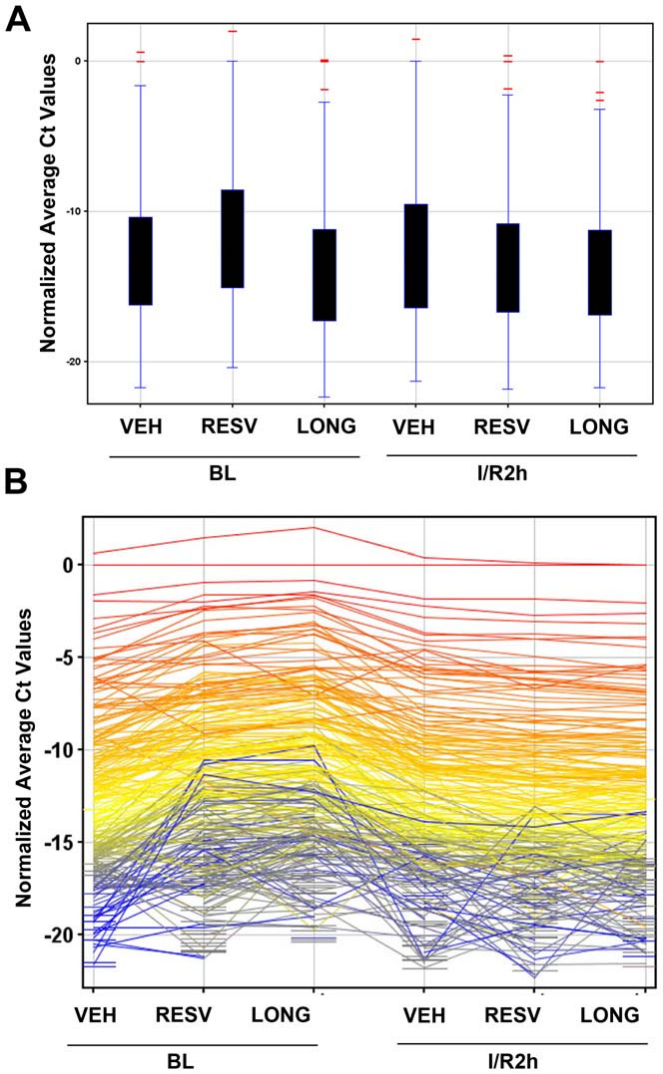
Effects of resveratrol and longevinex on global miRNA expression. (A) Box-Whisker Plot of miRNA array where outliers were shown outside the black bar and excluded for analysis. The data (Ct values) were normalized based on endogeneous genes. (B) Profile plot of miRNA array filtered on expression (20.0–100.0)th Percentile in the raw data which were normalized based on endogeneous genes. BL: Baseline; I/R2h: Ischemia for 30 min and 2 h reperfusion; VEH: Vehicle, RESV: Resveratrol; LONG: Longevinex.

**Figure 3:**
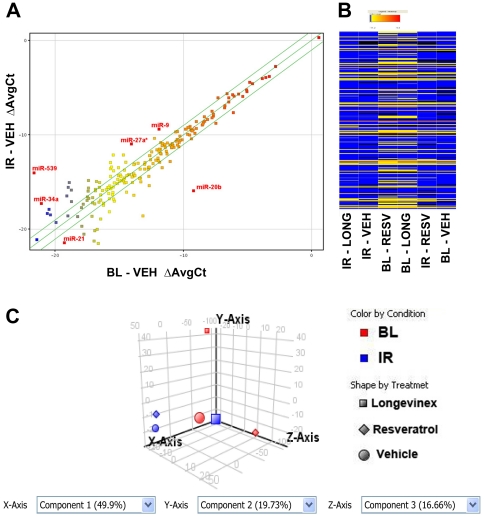
Effects of resveratrol and longevinex on miRNA expression pattern. (A) Correlation of miRNA expressions between basal level and IR control heart using scatter plot: Few miRNA expressions were selected for display as shown in table 1. (B) Heatmap for cluster analyses of differentially expressed miRNA among samples: Each miRNA was represented as single bar based from their Ct values and color coding was shown below with a gradient from blue (negative and lowest Ct values) to red (positive and highest Ct values). miRNAs not detected were shown as black bars. Each column was represented sample indicated on top. (C) Principal component analyses of all samples. This multivariate analysis demonstrated the proximity of longivinex and resveratrol treated IR samples to the control (vehicle) samples. BL: Baseline; IR: Ischemia for 30 min and 2 h reperfusion; VEH: Vehicle, RESV: Resveratrol; LONG: Longevinex.

MicroRNA profiles were analyzed by TLDA array specific for 586 miRNA and five endogeneous control for rat. Array were carried out in six different groups namely basal level (BL): (1) Vehicle, (2) Resveratrol, (3) Longevinex, and ischemic repurfused (IR): (4) Vehicle I/R, (5) pretreated (21days) with Resveratrol I/R and (5) pretreated (21days) with Longevinex I/R. RNAs were isolated after 30 min ischemia and 2 hour repurfusion of the heart from IR samples or from baseline (BL) samples processed the same way without ischemia and repurfusion. Box Whisker plot demonstrated unique distribution of total miRNA expression for all samples. The plot shows the median in the middle of the box, the 25th percentile (the lower quartile) and the 75th percentile (the upper quartile) (Fig. 2A). The whiskers are extensions of the box, snapped to the point within 1.5 times the interquartile. The points outside the whiskers were plotted and considered as the outliers. Few miRNAs were observed to be outliers and 385 miRNAs out of 586 were observed to be expressed at least in one of the six conditions. Expressions of 385 miRNAs were shown by profile plot after normalization to endogeneous control for each samples (Fig. 2B). miRNA expression were further analyzed by transforming to “fold change” compared to basal level control sample. Expressions of 213 miRNAs were expressed at least 2 fold or higher under one of six conditions. The list was further filtered after looking into miRNA which were either up or down 2-fold in IR samples. Top 25 miRNAs were listed, which were either up or down regulated in IR condition (Table 1) and the most regulations were reversed by pretreatment with resveratrol and longevinex. IR samples pretreated with resveratrol and longevinex both reversed the up or down regulation in IR Control in the opposite direction in 11 of the 25 miRNAs listed in Table 1. Either resveratrol or longevinex, but not both, reversed the up or down regulation compared to IR control in 5 instances. In rest of 9 miRNAs expression were attenuated by either or both. Longevinex exceeded the effect of resveratrol in 15 of the 25 miRNAs including miR-10a, miR-20b, miR-21. However, in few miRNAs such as miR-29c, longevinex have opposing effect to resveratrol and the difference may be due to many possibilities including presence of other ingredients in commercial formulation, bioavailability of resveratrol etc. There was a tremendous upregulation of miR-21 expression in basal level controls with resveratrol (up 391.4) and longevinex (760.9) which was lowered considerably in IR (up 61.5 and 59.3). miR-539 is upregulated to high level (214 fold) in IR samples and were further up-regulated in resveratrol preteated samples. Similar observation were also found in miR-27a, miR-101, miR-9, miR-667. Similar but less pronounced change were also found in many other miRNAs. Selected miRNAs were also shown in the scatter plot (Fig. 3A), where double lines indicated as fold change of 2.

**Table 1:** Differential expression of microRNA expressed in fold change with respect to basal level control heart sample.

miRNA	BL Resveratrol	BL Longevinex	IR Control	IR Resveratrol	IR Longevinex
miR-539	up 1272.9	up 642.7	**up 214.3**	up 172.4	up 314.6
miR-27a	up 2.2	up 2.1	**up 9.3**	up 5.5	up 1.4
miR-101a	up 28.4	up 39.2	**up 6.1**	up 3.1	up 3.3
miR-9	up 2.6	up 1.1	**up 5.4**	down 1.7	down 1.1
miR-667	up 8.2	up 6.3	**up 4.4**	up 2	up 1.2
miR-339-5p	up 13.6	up 20.7	**up 4.1**	down 1.4	down 3.8
rno-miR-345-3p	up 40.8	up 23.1	**up 3.7**	down 12	down 1.1
miR-10a	up 6.4	up 5.2	**up 3.5**	down 116	down 1.6
snoRNA202	up 3.8	up 4.7	**up 3.2**	down 6	down 3
miR-27b	down 1.4	up 1.9	**up 3.2**	up 1	up 1
miR-29c	up 5.4	up 4.5	**up 3.1**	up 1.5	down 1.5
miR-345-5p	up 14.3	up 31.7	**up 2.4**	down 4.7	up 1.1
rno-miR-24-1	down 25.3	up 1.2	**up 2.1**	down 1.2	down 1.9
miR-687	up 3.8	up 1.8	**up 2**	down 1.7	down 11.5
miR-27a	up 34	up 12.8	**up 1.6**	down 1.7	up 1.5
miR-31	up 2.4	up 1.1	**up 1.6**	down 17.5	down 2.1
miR-20b	down 6	down 38.8	**down 112.9**	down 189	down 1366
miR-760	down 2.7	up 2.5	**down 30.8**	up 1.5	up 2.2
miR-351	up 3.9	up 9.1	**down 20.9**	down 1.3	up 1.9
miR-181c	up 5.3	up 4.2	**down 6.7**	up 1.4	down 9.1
miR-21	up 391.4	up 760.9	**down 4**	up 61.5	up 59.3
miR-25	up 25	up 11.5	**down 1.9**	up 1.1	up 4.2
rno-miR-450a	up 4.8	up 2.4	**down 1.7**	down 1.5	down 5.4
miR-214	up 4.2	up 6.2	**down 1.3**	down 3.9	down 6.5
miR-324-3p	up 4.9	up 6.5	**down 1.2**	down 5.6	down 5.3

Top 25 miRNA were listed based its up or down regulation in IR samples.

**Figure 4:**
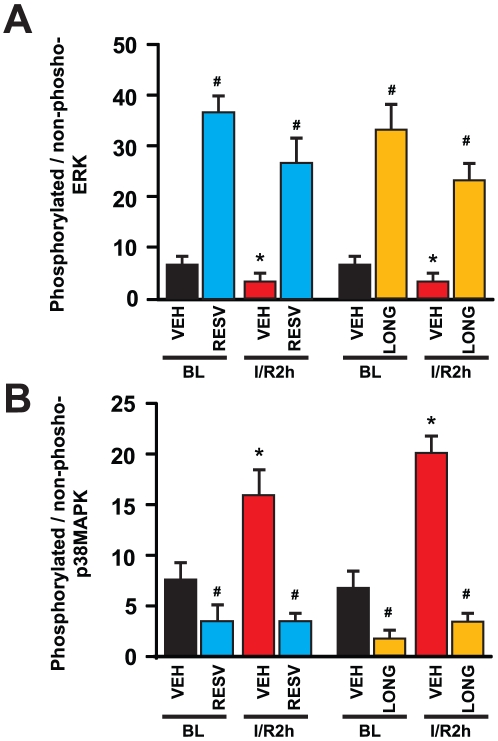
Effects of resveratrol and longevinex on phosphorylation of ERK1/2 and p38 MAPK. (A) Ratio of ERK1/2 phosphorylation to total ERK1/2 were plotted in samples as indicated. (B) Ratio of p38 MAPK phosphorylation to total p38 MAPK were plotted in samples as described. Results are expressed as Means±SEM of six animals per group. *p<0.05 vs. Vehicle (VEH). # p<0.05 vs corresponding I/R. BL: Baseline; I/R2h: Ischemia for 30 min and 2 h reperfusion; RESV: Resveratrol; LONG: Longevinex.

miR-539, the highest upregulated miRNA have 271 conserved gene target however functional target has not been reported in the literature. The targets of miR-539 obtained by computational analyses include matrix metallopeptidase 20, fibroblast growth factor 14, clathrin, light polypeptide, osteoprotegerin and transcription factors like forkhead box B1, which may have roles in cardiac remodeling. miR-21 were shown to regulate the ERK-MAP kinase signalling pathway in cardiac fibroblasts, which has role on global cardiac structure and function [24]. It has been also shown earlier that resveratrol triggers MAPK signaling pathway as a preconditioning mechanism in heart [25]. We also looked in samples the ERK-MAPK pathway. ERK phosphorylation was observed to be increased in both resveratrol and longevinex treated baseline samples and reduced in corresponding IR samples (Fig. 4A). Similar but opposing effect observed in p38 phosphorylation where significantly less phosphorylation occurred in resveratrol or longevinex treated BL samples. Increased p38 MAPK phosphorylation occurred in I/R2h samples and attenuated in both resveratrol and longevinex treated I/R2h samples due to preconditioning (Fig. 4B). VEGF is modulated by miR-20b through HIF1α in cardiomycytes whereas FOXO1 is regulated by miR-27a in cancer cells [26], [27]. SIRT1 were observed to be regulated by miR-9 in stem cells [28]. Recent studies demonstrated the increase of miR-1 in coronary artery diseases (CAD) and miR-1 is downregulated by beta-blocker propranolol in rat model of myocardial infarction [29]. Specific modulations of microRNA by resveratrol have not shown in any *in vivo* models. Recently microarray analysis of the effect of resveratrol has been demonstrated in human acute monocytic leukemia cell line (THP-1) and human colon adenocarcinoma cell line (SW480) [30], [31]. Resveratrol decreases the levels of miR-155 in THP-1 and modulating JunB and JunD, key regulators in carcinogenesis [31]. Resveratrol also modulates microRNA targeting effectors of TGFbeta pathways [30]. Treatment with resveratrol in cancer cell line SW480 results in decreased level of miR-21 and miR29c whereas it was increased in healthy heart when treated with resveratrol. This anomaly may be due to the fact that cardiomyocytes is barely dividing cells whereas SW480 cells grow rapidly which leads to complete different microenvironment inside cells. It is also important to point out that the doses for resveratrol is much higher (50micromolar) in cancer cells and similar dose is partially detrimental to human cardiomyocytes and endothelial cells in cultures (data not shown). Birds-eye-view of overall mapping of expression values were depicted by heatmap, a color-bar created by the range of values in the conditions of the interpretation (Fig. 3B). The expression value of each gene is mapped to a color-intensity value. It is evident from heat map that treatment with either resveratrol or longevinex in control samples altered significant miRNA expression levels, some of them may play significant key roles in cardio-protection.

### IR hearts pre-treated with resveratrol and longevinex closely related to miRNA pattern of BL control

Principal component analyses of the six samples revealed that the samples IR longevinex and IR resveratrol were remarkably similar to BL vehicle sample in terms of gene expression (Fig. 3C). In the majority of cases, they also were readily distinguished from each group. These results are indeed of utmost importance, as they document that both resveratrol and longevinex can protect the ischemic heart by restoring the IR-induced up-regulation or down-regulation of gene expression.

### Integrative analyses of miRNA for target gene and pathway analyses

Differentially expressed miRNAs were further analyzed for their putative target genes using TargetScan and were listed in the table 2. Most of the target genes(>1400 genes) have molecular function of metal ion binding, calcium-potassium-chloride ion binding, correlated to the restructuring heart after IR damage. Importantly, miRNA target gene modulated sequence specific DNA factor such as FOXO1, TRAF3 etc. SirT1 regulates several transcription factors including FoxO1, which is inactivated by phosphorylation via Akt [32]. Recent publication showed the phosphorylation of FoxO1 along with the activation of SirT1, SirT3 and SirT4 are localized in mitochondria where they regulate aging and energy metabolism.[4] Over the years,SIRT1 was known to be activated by resveratrol [33]. However, resveratrol may have no direct roles in activating SIRT1 [34]. Since dysregulation of miRNAs such as miR-21 is directly linked with cardiac diseases like ischemic heart disease and since resveratrol can ameliorate myocardial ischemic reperfusion injury through the modulation of several miRNAs, the results of the present study explains the mechanism of complex regulatory network mediated by resveratrol through miRNA in cardioprotection.

**Table 2:** Putative target genes for differentially expressed miRNA.

Molecular Function Category	Number of Target Genes	Example of Target Genes
RNA binding	101	Snrpe, Cherp,Phax
Actin binding	40	Tnni1, Cald1, Cfl1
Signal transducer activity	10	Gnb1, Wnt16
Receptor activity	55	Gpr155,Mmd2,Gab2
Structural molecule activity	31	Lmnb1,Krt1
Calcium ion binding	109	Ocm, Calm1, Rad21
Oxidoreductase activity	52	Duox2, Aldh2,Gpx7
Phosphatase activity	51	Mtmr1, Ptpn1,Styx
Potassium ion binding	50	Kcnc1, Slc12a4
Sodium ion binding	54	Scn4a, Hcn1
Chloride ion binding	40	Ano1, Ano1
Sequence-specific DNA binding	186	Foxo1, Traf3, Dnmt3b
Metal ion binding	1237	Dnmt3b, Rarb,Kcnd1

In summary, microRNA regulate target gene mostly by translational repression and sometimes through translational activation. Here, we demonstrated that resveratrol or longevinex regulated miRNA expression in healthy heart and ischemic-repurfused heart. Future detailed studies based on these analyses will pave the way for development of novel therapeutic intervention for cardioprotection in actute I/R injury.

## Methods

### Animals

All animals used in this study received humane care in compliance with the regulations relating to animals and experiments involving animals and adheres to principles stated in the Guide for the Care and Use of Laboratory Animals, NIH Publication, 1996 edition, and all the protocols (Proposal # 2008-484) were approved by the Institutional Animal Care Committee of University of Connecticut Health Center, Farmington, CT, USA. Male Sprague–Dawley rats weighing between 250 and 300 g were fed ad libitum regular rat chow with free access to water until the start of the experimental procedure. Animals were gavaged with either resveratrol (5 mg/kg/day) [Sigma Chemical Company, St. Louis, MO] or longevinex (100 mg/kg/day) [Longevinex Inc, North Las Vegas, NV] for 21 days. Previous studies from our laboratory established the appropriate dose and time periods for each compound used in this experiment [35], [36].

### Isolated working heart preparation and assessment of cardiac function

After completing the feeding protocol, the animals were anesthetized with sodium pentobarbital (80 mg/kg, i.p.) (Abbott Laboratories, North Chicago, IL, USA), and intraperitonealy heparin sodium (500 IU/kg, i.v.) (Elkins-Sinn Inc., Cherry Hill, NJ, USA) was used as an anticoagulant. After the deep anesthesia was conformed, hearts were excised, the aorta was canulated, and the hearts were perfused through the aorta in Langendorff mode at a constant (100 cm of water) perfusion pressure at 37°C with the KHB for a 5 min washout period as described previously. The perfusion medium consisted of a modified Krebs-Henseleit bicarbonate buffer (millimolar concentration: sodium chloride 118, potassium chloride 4.7, calcium chloride 1.7, sodium bicarbonate 25, potassium dihydrogen phosphate 0.36, magnesium sulfate 1.2 and glucose 10), and after its oxygenization pH was 7.4 at 37°C. During the washout period left atria was canulated, and the Langendorff preparation was switched to the working mode for 10 min with a left atrial 6 filling pressure of 17 cm H_2_O, aortic afterload pressure was set to 100 cm of water. At the end of 10 min, baseline cardiac function like heart rate (HR, beats/min), aortic flow (AF, ml/min), coronary flow (CF, ml/min), left ventricular developed pressure (LVDP, mmHg) and first derivative of developed pressure (LVdp/dt, mmHg/sec) were recorded. After that 30 min of global ischemia was initiated by clamping the left atrial inflow and aortic outflow lines at a point close to their origins. At the end of the 30 min of ischemia, reperfusion was initiated for 60 min or 120 min by unclamping the atrial inflow and aortic outflow lines. The first 10 min reperfusion was in Langendorff mode to avoid the ventricular fibrillations, after the hearts were switched to anterograde working mode [4].

### Infarct size estimation

Infarct size was measured according to the TTC method [4], [7]. After the 2 h of reperfusion, 40 ml of 1% (w/v) solution of triphenyl tetrazolium chloride (TTC) in phosphate buffer was infused into aortic cannula, and the heart samples were stored at -70°C for subsequent analysis. Sections (0.8 mm) of frozen heart were fixed in 2% paraformaldehyde, placed between two cover slips and digitally imaged using a Microtek ScanMaker 600z. To quantitate the areas of infarct in pixels, standard NIH image program was used. The infarct size was quantified and expressed in pixels [4], [7].

### Assessment of apoptotic cell death

Immunohistochemical detection of apoptotic cells was carried out using the TUNEL method [4], [7] (Promega, Madison, WI). Briefly, after the isolated heart experiments the heart tissues were immediately put in 10% formalin and fixed in an automatic tissue fixing machine. The TUNEL staining was performed according to the manufacturer's instructions. The fluorescence staining was viewed with a fluorescence microscope (AXIOPLAN2 IMAGING, Carl Zeiss Microimaging Inc., New York) at 520±20 nm for green fluorescence of fluorescein and at 620 nm for red fluorescence of propidium iodide. The number of apoptotic cells was counted and expressed as a percent of total myocyte population.

### Micro RNA isolation and cDNA preparation

Total RNA from rat heart samples were isolated using Trizol reagent (Invitrogen) and further purified using mirVANA miRNA isolation kit (Ambion) [37]. cDNAs were prepared using Taqman miRNA Reverse Transcription kit and Megaplex Rodent Pool A and B primers sets.

### Profiling of miRNA expression

miRNA expression profiling were carried out using quantitative real-time PCR method by TaqMan® Gene Signature Rodent Arrays on a 384 well micro fluidic card in 7900HT Realtime PCR machine(Applied Biosystem, Foster City) according to manufacturer's recommendation. Each miRNA were quantified by two specific amplicon primers and one specific probe. Comprehensive coverage of Sanger miRBase v10 was enabled across a two-card set of TaqMan® MicroRNA Low Density Arrays (TLDA Array A and B) for a total of 518, and 303 unique assays, specific to rat miRNAs, respectively. In addition, each array contains six control assays—five carefully selected candidate endogenous control assays, and one negative control assay. Profiling of miRNA by array has been used previously [38].

### Analyses of miRNA gene expression data

Realtime PCR data expressed as Ct values from array A and B were combined using R script (provided by GeneSpring Informatics Support Team) and processed using GeneSpringGX 11.0.2 software (Agilent Technologies, Santa Clara). After analysis 591 entities were detected from array A and B. All statistical analyses including normalization to endogeneous control, quality control, filtering, correlation analyses and principal component analyses were carried out by GeneSpring GX software.

### miRNA Target prediction

miRNA targets have been predicted using TargetScan in-built and plugged within GeneSpring GX software.

### Western Blot analysis

Hearts were homogenized in a buffer containing 25 mM Tris-HCl, 25 mM NaCl, 1 mM orthovanadate, 10 mM NaF, 10 mM pyrophosphate, 10 µM okadaic acid, 0.5 mM EDTA, and 1 mM phenylmethylsulfonyl fluoride. One hundred micrograms protein of each heart homogenates separated by SDS-polyacrylamide gel electrophoresis and immobilized on polyvinylidene difluoride membrane. The membrane was immune-blotted with ERK1/2, phospho-ERK1/2, p38 MAPK and phospho-p38 MAPK (Cell signaling Technology, MA) to evaluate the phosphorylation of the compounds. The resulting blots were digitized and subjected to densitometric scanning using a standard NIH image program.

### Statistical analysis

The values for myocardial function parameters, infarct size and apoptosis were expressed as the mean ± standard error of mean (SEM). A one-way analysis of variance was first carried out to test for any differences in mean values between groups. If differences were established, the values of the resveratrol-treated groups were compared with those of the control group by modified t-test. The results were considered significant if p<0.05.
